# Calculation of the pharmacogenomics benefit score for patients with medication-related problems

**DOI:** 10.3389/fgene.2023.1152585

**Published:** 2023-05-05

**Authors:** Thomas M. Polasek

**Affiliations:** ^1^ Certara, Princeton, NJ, United States; ^2^ Centre for Medicines Use and Safety, Monash University, Melbourne, VIC, Australia

**Keywords:** pharmacogenomics benefit score, pharmacogenomics, precision medicine, precision dosing, adverse drug reactions

## Abstract

Unexpected poor efficacy and intolerable adverse effects are medication-related problems that may result from genetic variation in genes encoding key proteins involved in pharmacokinetics or pharmacodynamics. Pharmacogenomic (PGx) testing can be used in medical practice “pre-emptively” to avoid future patient harm from medications and “reactively” to diagnose medication-related problems following their occurrence. A structured approach to PGx consulting is proposed to calculate the pharmacogenomics benefit score (PGxBS), a patient-centered objective measure of congruency between medication-related problems and patient genotypes. An example case of poor efficacy with multiple medications is presented, together with comments on the potential benefits and limitations of using the PGxBS in medical practice.

## Background

There is growing interest in using pharmacogenomics (PGx) broadly in medical practice to improve the chances of therapeutic success in individual patients by precision dosing (Polasek et al., 2018; Polasek et al., 2019). Clinical guidelines are available to instruct doctors on how to prescribe select medications based on patient genotypes (Relling and Klein, 2011). Ideally, this should be done prior to commencing treatment, which is called “pre-emptive” PGx testing. There are many examples in well-resourced healthcare systems of PGx services being implemented successfully, usually via electronic clinical decision support systems (CDSS) (Dunnenberger et al., 2016); patients are screened and almost all (>95%) are found to have genetic variants with so-called “actionable PGx guideline recommendations” that could influence future prescribing (Mostafa et al., 2019). Less frequently addressed in the PGx literature is the clinical scenario where patients have histories of medication-related problems at standard doses without an obvious explanation, either unexpected poor efficacy or intolerable adverse effects. “Reactive” PGx testing can be used in these patients to diagnose whether PGx is the potential cause. Pharmacogenomic testing is therefore a unique pathology test that has dual clinical utility depending on when the test is ordered and/or reviewed relative to the medication prescribed i.e., a screening test to avoid future patient harm and a diagnostic test in the work-up of differential diagnoses. Whilst there is growing evidence for pre-emptive PGx testing to decrease adverse drug reactions (ADRs), by as much as 30% in some studies (Zhou et al., 2015; Cacabelos et al., 2019; Swen et al., 2023), the degree to which reactive PGx testing diagnoses the cause of medication-related problems is unclear.

In this report, a structured approach to PGx consulting by a clinical pharmacologist is described based on referrals of patients with current and/or past medication-related problems (Aronson, 2010). The pharmacogenomics benefit score (PGxBS) is proposed as a patient-centered objective measure of congruency between medication-related problems and patient genotypes. An example case of unexpected poor efficacy with multiple medications is presented to show how the PGxBS is calculated. Finally, consideration is given to the potential benefits and limitations of using the PGxBS in medical practice.

## Categories of PGx

There are three categories to consider when diagnosing PGx as the potential cause of medication-related problems.

**1) Exposure PGx.** *Is the patient at risk of extreme exposure to the medication at standard doses?* Pharmacokinetic processes determine “how much” a medication is available at the sites of action, and therefore, assuming typical dose-exposure-response relationships, the magnitude of response. Extremely high medication exposures are associated with an increased risk of adverse effects, whereas persistently low medication exposures may result in subtherapeutic concentrations and poor efficacy. Although many genes influence pharmacokinetics, the cytochrome P450 (CYP) enzymes are the most important for PGx (Doogue and Polasek, 2013).

**2) Response PGx.** *Does the patient have the correct molecular target for the medication?* At a given exposure, genetic variability in the molecular target can determine the response (pharmacodynamics). This is best exemplified currently in hematology and oncology; patients are treated with targeted pharmacotherapy based on the results of genetic testing of the molecular targets expressed by cancer cells (Polasek et al., 2016). This category will expand in the future as genomic analyses identify novel pharmacodynamic biomarkers of response (Dawed et al., 2023).

**3) Safety PGx.** *Is the patient at risk of a severe adverse drug reaction to the medication at standard doses?* There is some overlap here with category 1 (Exposure PGx) and category 2 (Response PGx) but this category primarily includes rare severe cutaneous adverse drug reactions (SCARs) in patients with certain human leukocyte antigen (HLA) genotypes. In these cases, the patients’ immune system carries genetic variants that significantly increase the likelihood of ADRs (Kloypan et al., 2021).

## Structured PGx consult

**FIGURE 1:**
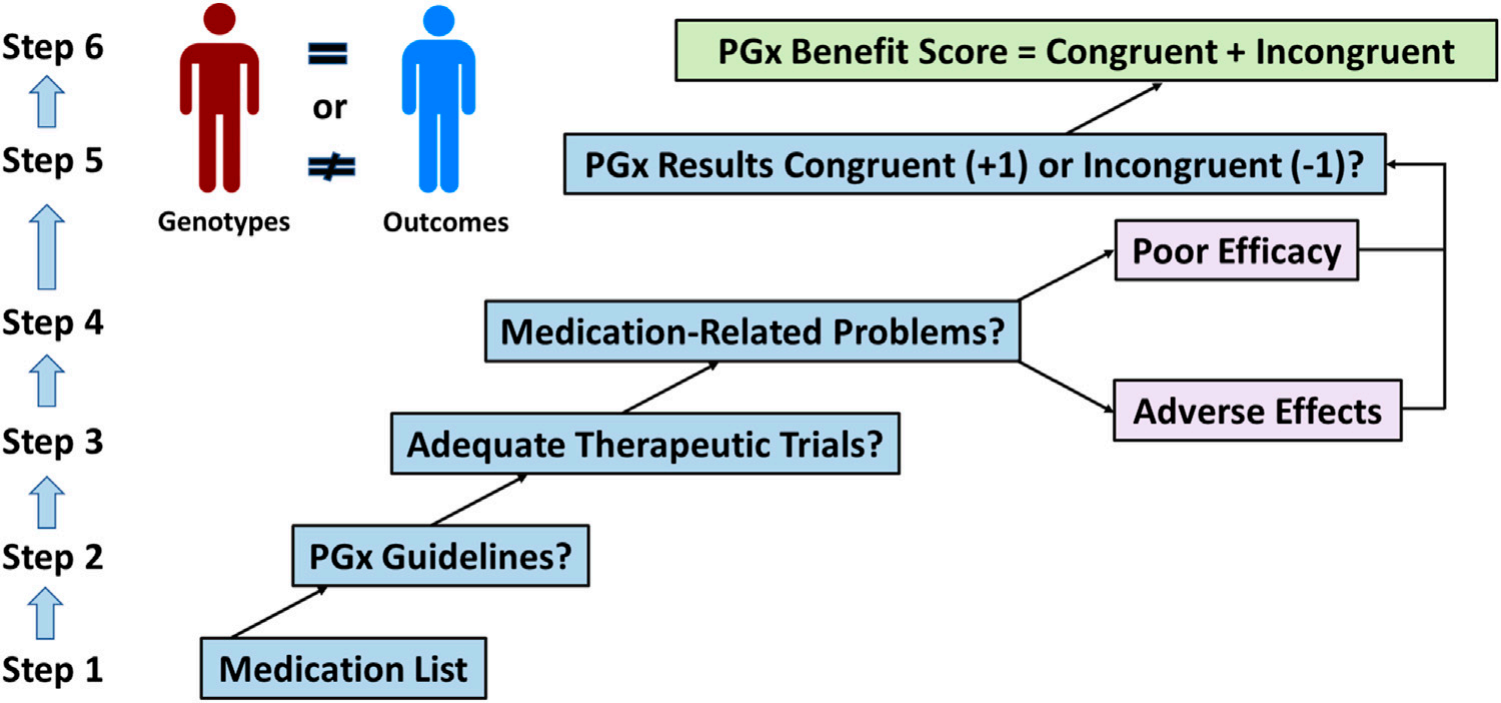
Structured approach to PGx consulting for medication-related problems.

A structured approach to PGx consulting by a doctor is suggested here because medication-related problems should be considered under differential diagnoses. This requires diagnostic skill and experience, and a broader understanding of the patient beyond simply medications and genotypes (Aronson, 2010) (Figure 1). The doctor may or may not have access to an electronic CDSS with PGx guidance (Wake et al., 2021). The following steps outline the information required to calculate the PGxBS. Binary responses to the main steps are required. A spreadsheet can be used to log answers and calculate scores.

**1) List current and past medications.** Current medications have priority. Past medications are also important to capture if time permits, since clues on how patients respond to medications more broadly may be garnered, further informing the PGxBS.

**2) Determine availability of PGx guidelines.** For each medication, determine whether Clinical Pharmacogenomics Implementation Consortium (CPIC^®^) level A or A/B evidence is available (www.cpicpgx.org). Assign one if the answer is “yes” and 0 for “no/unsure”. If there are no medications with PGx guidelines, then the PGx consult is complete and the PGxBS for the patient is 0.

**3) Assess adequacy of therapeutic trials.** For each medication with PGx guidelines, determine whether the patient had an adequate therapeutic trial or not. Assign one if the answer is “yes” and 0 for “no/unsure”. Inadequate therapeutic trials from underdosing or short durations of treatment are common and should be recognised, scoring 0. This section can also be completed for medications without PGx guidelines to improve the medication history, but these responses do not count towards the PGxBS.

**4) Determine therapeutic outcomes.** Two types of medication-related problems indicate negative therapeutic outcomes that could be explained by PGx—unexpected poor efficacy or intolerable adverse effects (Polasek et al., 2018). One is chosen here, scoring 1, with the alternative scoring 0. Medications with inadequate therapeutic trials (step 3) are ignored.

**5) Determine congruency between therapeutic outcomes and PGx results.** Is each medication-related problem consistent with the genotype-predicted phenotype? Again, this is a binary option, with congruent results scoring 1 and incongruent results scoring -1. The same PGx guidelines from step 2 (CPIC^®^) should be used. An example of a congruent result is a patient who experienced SCAR after starting allopurinol and who was subsequently shown to carry the HLA–B*5801 allele (Lucas and Droney, 2022). Alternatively, a chronic pain sufferer with a CYP2D6 poor metabolizer (PM) phenotype who experienced euphoria and intolerable dizziness and nausea with low dose tramadol is an example of an incongruent result (Crews et al., 2021).

**6) Calculate the PGxBS.** Congruent and incongruent results are added. Scores ≥1 indicate a possible contribution of PGx to medication-related problems, whereas 0 and negative scores show that PGx is less likely to be important for the patient.

## Example case

**FIGURE 2:**
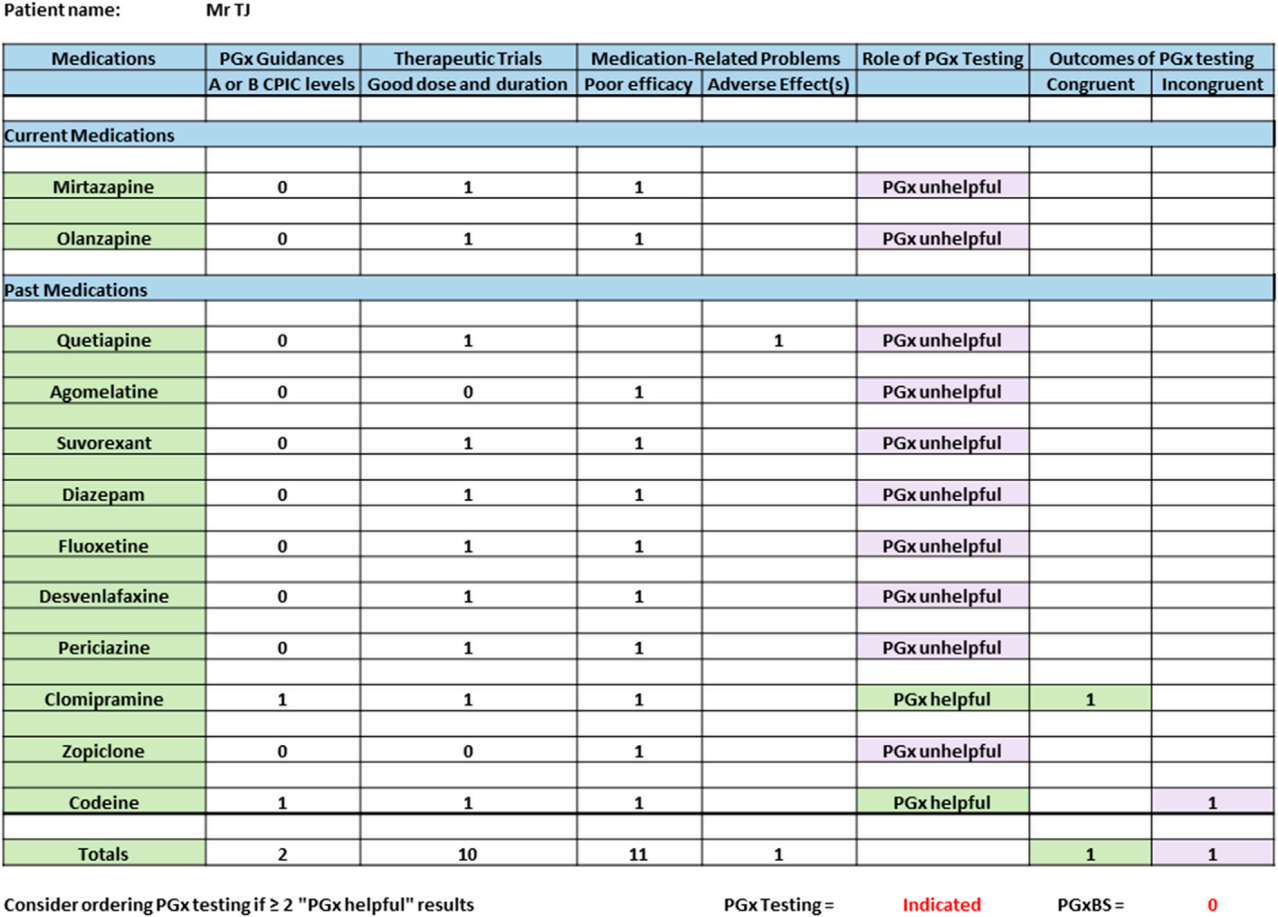
Example case showing how to calculate the PGx benefit score.

A 42-year-old man with a 6-year history of depression, anxiety, insomnia, and chronic lower back pain was referred by his general practitioner to a multi-disciplinary ambulatory care clinic staffed by clinical pharmacologists for “poor responses to psychotropics and pain killers”. His mental state had deteriorated over the previous 3 months, and he was awaiting psychiatrist review. Figure 2 shows the spreadsheet used to document the consult and calculate his PGxBS. Since the patient had two medications with PGx guidelines and no previous PGx testing, it was recommended, and the patient accepted the cost (∼$100USD). The PGx results were reconciled with the medication-related problems at the follow-up appointment. His CYP2D6 ultra-rapid (UM) metabolizer phenotype was incongruent with poor analgesic response to codeine (score = -1). However, there was congruency between CYP2D6 UM and CYP2C19 normal metabolizer (NM) phenotypes and no improvement in mental state with clomipramine (score = 1). The PGxBS was 0. Importantly, this patient held strong beliefs about being “abnormal” and “unable to be helped by drugs”. Counselling was provided to explain that no known genetic cause for his poor responses was found. The patient was encouraged to be positive about medications in his overall treatment. The PGx spreadsheet was included in the medical consult note and forwarded to his treating general practitioner and psychiatrist (Figure 2).

## Potential benefits of the PGxBS

The PGxBS is a clinically useful objective measure of congruency between medication-related problems and patient genotypes. The score is patient-centered rather than focused on individual medication-gene pairs (“this is your PGxBS”). The score is easy to understand for patients and non-expert PGx users—positive results indicate a possible role for PGx, whereas zero and negative scores mean that PGx is less likely to be important. The PGxBS may be applied to patients with single or multiple current and/or past medication-related problems. The PGxBS is dynamic and changes with time and changing medication regimens. Calculating a patient’s PGxBS requires particular attention to the medication history, which alone has benefits for clinical care. Importantly, the structured PGx consult allows for patient education on the many factors that explain why different patients respond to medications differently, including pharmacokinetic drug-drug interactions that cause CYP phenoconversion (Mostafa et al., 2021; Mostafa et al., 2022). Although the emphasis in this report is on medical practice, pharmacists with expertise in PGx could calculate the PGxBS and integrate it into their clinical practice, ideally in close collaboration with the treating doctor (Polasek et al., 2015).

## Limitations of the PGxBS

The PGxBS does not apply to pre-emptive PGx testing, where, at least in principle, almost all patients will benefit i.e., >95% have genetic variants with so-called “actionable PGx guideline recommendations” (Mostafa et al., 2019; Swen et al., 2023). In patients with medication-related problems who have not been tested, two or more medications with PGx guidelines is the suggested cut-off for reactive PGx testing. This is only a guide since the clinical need (indication) for reactive PGx testing depends on many factors, including disease status, differential diagnoses, severity of treatment outcomes, treatment alternatives, and test affordability. The PGxBS is not validated for clinical decision-making, including prescribing. To date, the score has not been applied beyond one clinical pharmacology referral stream in Australia. Whether a patient’s present score reflects the future clinical utility of PGx for that patient is unknown. The PGxBS often depends on the recollection of subjective past experiences with medications, occurring years previously in some cases, and there may be intrinsic biases. Finally, there are nuances to the PGxBS that are debatable, such as the PGx guidelines and levels of evidence chosen (step 2).

## Conclusion

Despite the promise of superior patient care and considerable academic and commercial interests, adoption of PGx in routine medical practice has been limited (Pearce et al., 2022). Whilst there is growing evidence for pre-emptive PGx testing to avoid ADRs, the degree to which reactive PGx testing diagnoses the cause of medication-related problems is less clear. Rather than details about individual medication-gene pairs, patients with histories of medication-related problems and their doctors are often more interested in whether PGx is “the answer”. In such cases, a structured approach to PGx consulting is recommended to generate the PGxBS, a patient-centered objective measure of congruency between medication-related problems and patient genotypes.

## Data Availability

The original contributions presented in the study are included in the article/Supplementary Material, further inquiries can be directed to the corresponding author.
